# In Vivo Safety Evaluation of Acoustic Radiation Force for Optical Coherence Elastography of the Crystalline Lens

**DOI:** 10.1167/tvst.14.8.35

**Published:** 2025-08-26

**Authors:** Leana Rohman, Juan-Carlos Navia, Christian Zevallos-Delgado, Heather Durkee, Justin Schumacher, Karna Nagalla, Natalie Zaleski, Marco Ruggeri, Manmohan Singh, Salavat R. Aglyamov, Jean-Marie Parel, Giuliano Scarcelli, Kirill V. Larin, Fabrice Manns

**Affiliations:** 1 Ophthalmic Biophysics Center, Bascom Palmer Eye Institute, University of Miami School of Medicine, Miami, FL, USA; 2 Department of Biomedical Engineering, University of Miami College of Engineering, Coral Gables, FL, USA; 3 Department of Biomedical Engineering, University of Houston, Houston, TX, USA; 4 Department of Bioengineering, University of Maryland, 8278 Paint Branch Drive, College Park, MD, USA; 5 Department of Mechanical and Aerospace Engineering, University of Houston, Houston, TX, USA

**Keywords:** acoustic radiation force (ARF), safety, in vivo lens biomechanics, optical coherence elastography (OCE), Brillouin microscopy, shear wave

## Abstract

**Purpose:**

To evaluate the safety of acoustic radiation force (ARF) for in vivo lens biomechanics measurement in rabbits.

**Methods:**

Twelve New Zealand albino white rabbits were exposed to acoustic radiation force at intensities exceeding the US Food and Drug Administration (FDA)–recommended safety limits by 8 to 14 times. A spherically focused 3.5-MHz ARF transducer created deformations on the lens surface, which was imaged using a spectral-domain optical coherence tomography system during the ARF application. Intraocular pressure measurements and ocular health assessments using slit-lamp and OCT imaging were conducted pre- and postexposure over 3 weeks.

**Results:**

Hyperemia was observed in two rabbits immediately postexposure but resolved within 24 hours. No substantial changes in intraocular pressure were detected, and both slit-lamp examination and optical coherence tomography imaging showed normal ocular health across all groups after the follow-up period.

**Conclusions:**

ARF is a potentially safe technique for assessing the biomechanical properties of the lens in vivo. No eye damage was observed, even when ARF was applied at intensities well above FDA regulatory limits.

**Translational Relevance:**

This study is an important step toward the translation of the technology for ARF elastography of the crystalline lens, for studies on the mechanism of presbyopia, and to enable the assessment of new presbyopia treatments relying on lens softening.

## Introduction

There is strong evidence that lens stiffening with age is a major factor in the development of presbyopia.^1^^–^^3^ Based on this finding, there have been efforts to develop lens-softening treatments using pharmaceuticals or lasers.^4^^,^^5^ To determine the effectiveness of these treatments, a technique to measure the mechanical properties of the lens in the intact eye globe is required. Traditionally, investigations into the lens's mechanical properties have been conducted ex vivo, employing methods like lens spinning, compression, stretching, or dynamic mechanical analysis.^6^^–^^9^ In vivo measurements have been acquired using Brillouin microscopy, but Brillouin microscopy provides measurements at very high frequencies in the gigahertz range and measures the longitudinal modulus (i.e., compressibility), which is not directly related to Young's modulus (i.e., shear elasticity), measured in vitro and at relevant physiological scales. We have developed an approach for multimodal elastography by combining optical coherence elastography (OCE) and Brillouin microscopy to measure the mechanical properties of the lens.^10^ The feasibility of the approach was demonstrated in porcine eyes.^10^ Brillouin microscopy gives a relative estimation of the mechanical properties of the lens, while OCE will provide an absolute value measurement that will help calibrate the Brillouin measurements, enabling high-resolution mapping of lenticular stiffness.

OCE measures the mechanical properties of tissue by using optical coherence tomography (OCT) imaging to measure the deformation of tissue generally induced by an external force.^11^ For in vivo lens measurements, the external force must be transmitted through the cornea and aqueous humor to the lens surface. Acoustic radiation force (ARF) has been used to induce ocular tissue deformation for measurement of elasticity^12^^,^^13^ of the cornea,^14^ lens,^15^^,^^16^ and retinal layers.^17^^,^^18^ Research by Li et al.^13^ demonstrated the feasibility of using ARF excitation to deform the lens of live rabbits and assess its mechanical characteristics. However, these experiments were conducted at ARF intensities surpassing the US Food and Drug Administration's (FDA's) recommended limits for ophthalmic ultrasound: derated spatial peak temporal average intensity less than 50 mW·cm^−2^, thermal index (TI) less than 1, mechanical index (MI) less than 0.23, or derated spatial peak pulse average intensity (I_SPPA.3_) less than 28 W·cm^−2^.^19^ Li et al.^13^ used values that were 6.5 times above the mechanical index limit of 0.23. On the other hand, Zevallos-Delgado et al.^20^ recently showed that applying ARF excitation to whole ex vivo porcine eyes at the FDA guidelines produced no damage to the lens until intensities far exceeding the limits.

To date, only one study has explored the impact of ARF on eyes in vivo. In this research, Zha et al.^12^ used a commercial clinical ultrasound system (Acuson S2000; Siemens, Malvern, PA, USA) in impulse elastography mode to expose rat eyes to ARF impulses with a mechanical index of 1.6, which is seven times higher than the FDA limit of 0.23. The rats were followed for a period of 7 days. Even with this high peak power, they did not observe any detrimental effects and concluded that ARF is potentially safe for application in human eyes. However, the size and anatomy of the rat eye are very different from that of the human eye, and the study used a large linear transducer array designed for nonophthalmic use that measures the longitudinal displacement of tissue in response to an axial ARF in a wide region of interest, with axial displacement on the order of 20 µm.

The goal of the present study was to evaluate the feasibility and safety of ARF excitation of the lens in live rabbits. Our primary endpoints are to demonstrate the feasibility of applying ARF to the lens without inducing a cataract, anterior segment injury, or inflammation on clinical examination during a 3-week period following ARF application.

## Methods

We performed our safety evaluation on 12 female New Zealand albino white rabbits following the University of Miami's Institutional Animal Care and Use Committee–approved protocol and with adherence to the ARVO Animal Statement. Rabbit eyes were selected for their anatomic similarity to the human eye, enabling the use of ARF protocols mimicking human use. The right eye was exposed to ARF, while the left eye served as a control. All rabbits included in the study were female, aged 3 months or older, with an average weight of 3.3 ± 0.7 kg at the time of the ARF exposure. The rabbits were housed in an Association for Assessment and Accreditation of Laboratory Animal Care–certified animal care facility at the University of Miami. The rabbits were attended to and cared for by the University of Miami Division of Veterinary Resources (DVR) animal care staff. The animals’ diet consisted of hay, vegetables, fruits, and a specially formulated animal diet. All examinations and procedures were performed by an ophthalmologist who received training in the handling and care of rabbits from a veterinarian at the DVR.

The sample size in each group reflects a careful consideration of the minimal number of experiments required to obtain meaningful results, while minimizing the number of animals. The goal of the study is to demonstrate the feasibility and safety of applying ARF excitation to the lens. We estimated that a sample size of three animals per group would be sufficient to demonstrate safety, given that prior studies found no evidence of adverse effects at ARF levels well above the FDA guidelines.

**Figure 1.**
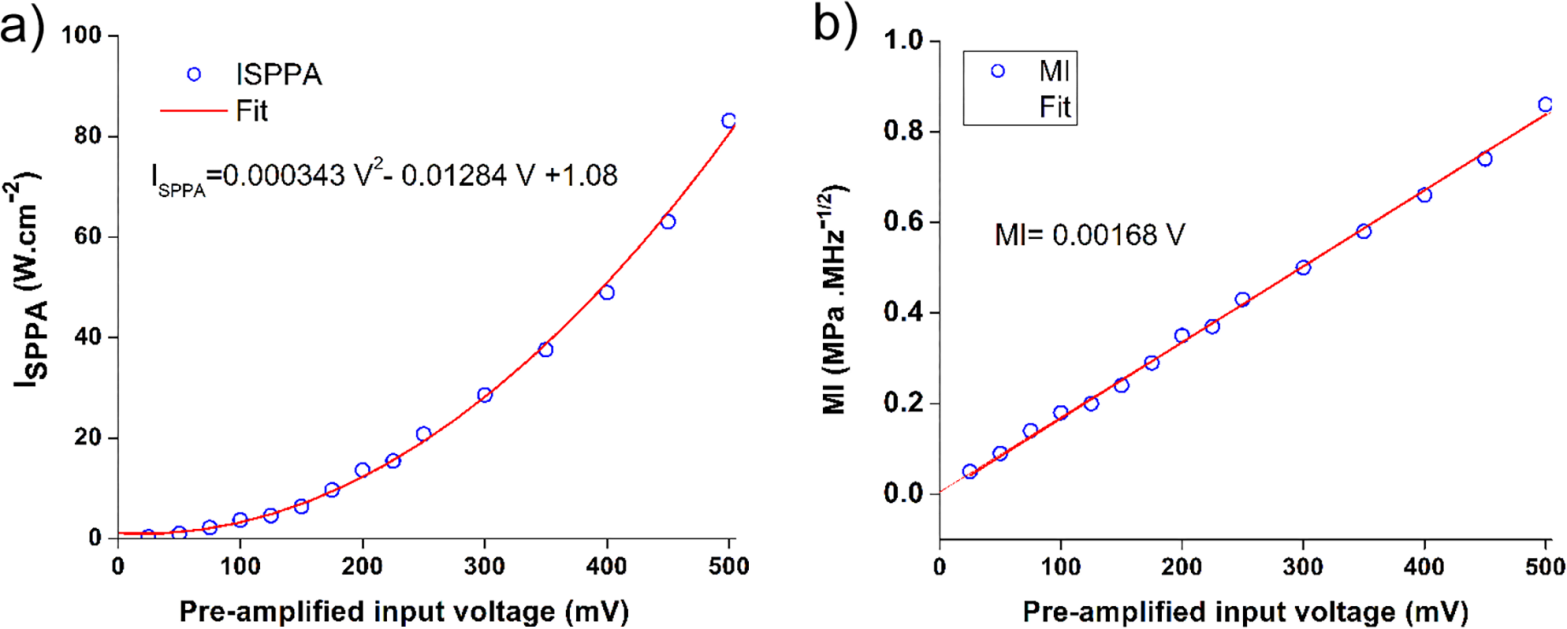
(**a**) Profile of spatial peak pulse average intensity and (**b**) mechanical index as a function of the preamplified input (mV).

To generate mechanical waves at the lens surface, we employed a spherically focused, single-element, 3.5-MHz ultrasound transducer (V382-SU; Olympus Corp., Waltham, Massachusetts, USA) with a focal length of 21 mm and a diameter of 13 mm. Consistent with our previous work,^15^^,^^20^^,^^21^ the transducer was calibrated using a needle hydrophone with a 0.2-mm sensor diameter (NH0200; Precision Acoustics Ltd, Dorchester, UK) across a range of preamplified voltages from 25 to 500 mV. This calibration provided the relationship between excitation voltage and generated acoustic pressure, as detailed in the Table and illustrated in Figure 1. Spatial peak pulse average intensity and mechanical index were calculated based on the acoustic pressure measurements. As shown in the Table, the FDA safety limits were met for a preamplified voltage below 250 mV for I_SPPA_ and below 150 mV for MI. Consequently, we selected a preamplified voltage of 145 mV as the reference voltage, at which MI equals the FDA limit of 0.23, and I_SPPA_ is approximately 20% of the limit, or around 5.5 W·cm^−2^. Four preamplified voltages were used: one, two, four, and eight times the reference voltage, corresponding to 145 mV, 289 mV, 578 mV, and 1145 mV, respectively. The estimated MI values for these voltages were approximately 0.23, 0.47, 0.95, and 2.02, while the I_SPPA_ values were 5.5, 22.5, 94, and 387 W·cm^−2^, respectively. Thus, at the maximum voltage, MI exceeded the FDA safety limit by approximately 8 times, while I_SPPA_ exceeded it by approximately 14 times. The voltage (eight times) was selected based on our prior in vitro study on porcine eyes,^20^ which found that there was no damage at exposure below 14 times the exposure limit for the MI. Since the present study involves survival for a period of 3 weeks, we selected an exposure level below the level that produced structural damage in vitro to minimize the risk of injury, pain, and distress.

**Table.** Ultrasound Transducer Acoustic Characteristics

Preamplified Input Peak Amplitude, mV	Hydrophone Peak Amplitude, mV	Pressure Peak Amplitude, MPa	I_SPPA_, W/cm^2^	MI
25	14.80	0.10	0.34	0.05
50	25.60	0.18	1.01	0.09
75	37.60	0.26	2.18	0.14
100	48.80	0.34	3.68	0.18
125	54.40	0.38	4.57	**0.20**
150	64.60	0.44	6.38	0.24
175	79.20	0.55	9.69	0.29
200	94.00	0.65	13.64	0.35
225	100.00	0.69	15.44	0.37
250	116.00	0.80	**20.78**	0.43
300	136.00	0.94	28.56	0.50
350	156.00	1.08	37.58	0.58
400	178.00	1.23	48.93	0.66
450	202.00	1.39	63.01	0.74
500	232.00	1.60	83.12	0.86

I_SPPA_ and MI values not exceeding FDA safety limits are in bold.

The rabbits were divided into four groups, each consisting of three rabbits treated at four different ARF levels: one, two, four, and eight times the reference voltage, using one intensity level per group. Three days before performing the experiments, a slit-lamp ophthalmic examination was performed, intraocular pressure (IOP) was measured with the iCare Tonovet (ICare, Vantaa, Finland) rebound tonometer under topical anesthesia (proparacaine hydrochloride ophthalmic solution 0.5%), and OCT images of the anterior segment were acquired on the awake animal to document baseline data before ARF exposure.

On the experiment day, first, the IOP was measured in both eyes with the same rebound tonometer. Using an aseptic technique, the rabbits were then placed under sedation and general anesthesia using a cocktail comprising 100 mg/mL xylazine, 10 mg/mL acepromazine maleate, and 100 mg/mL ketamine. The anesthetic dosages were calculated based on the rabbit weight according to the guidelines provided by the Division of Veterinary Resources at the University of Miami. Tropicamide 1% ophthalmic solution was administered to induce mydriasis. Once the rabbit was determined to be unconscious and in a sufficient depth of anesthesia by the absence of pedal withdrawal and corneal reflexes, it was transferred onto a transport tray and subsequently moved to the surgical table, where the pretreatment ophthalmic examination and the OCT were performed. Special attention was given to maintaining a clear airway for the rabbit's safety, monitoring cardiac frequency, respiration rate, and temperature every 15 minutes. The rabbit was covered with a blanket to prevent a drop in body temperature.

To observe the ARF-induced deformations in real time, we used a commercial research-grade spectral-domain optical coherence tomography (SD-OCT) system operating at 1300 nm (Thorlabs Telesto, Newton, NJ). To ensure precise alignment of the transducer with the OCT beam for inducing mechanical waves in the lens and subsequent observation, we developed a custom three-dimensional (3D) mount securely attached to the OCT objective that precisely coaligns the OCT and ARF beams’ focus. The OCT scanning system was attached to a custom-built motorized stage, which was remotely controlled to adjust the positioning of the objective vertically (up and down) and horizontally (left and right).

**Figure 2.**
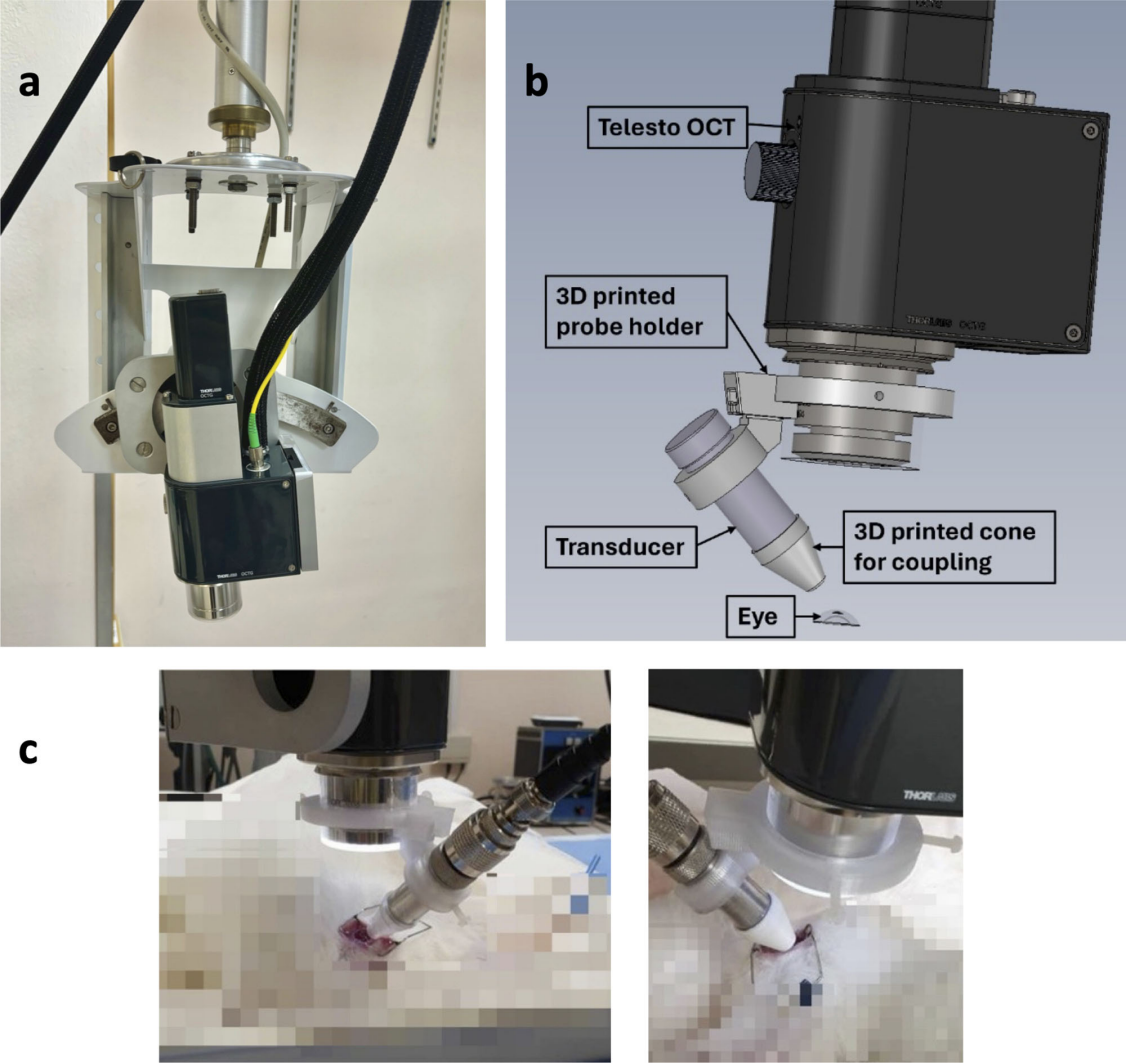
(**a**) OCT scanner mounted on a motorized arm. (**b**) Experimental setup with the 3D mount. (**c**) Experimental setup during the procedure.

We prepared the system by attaching a 3D-printed cone filled with ARF coupling gel to the head of the transducer for acoustic coupling to the eye. Then, the OCT delivery probe and ultrasound transducer, which were mounted on a motorized stage, were first manually positioned over the rabbit's eye. Subsequently, we used the remote control and the live OCT image acquisition window to precisely adjust the position of both the OCT probe and the transducer until the gel coming out of the cone tip was in contact with the cornea to optimize ARF transmission into the rabbit's lens. Figure 2 illustrates the experimental setup.

Once the OCT probe and ultrasound transducer were positioned, the transducer was activated to deliver ARF excitation. The pulse repetition frequency for the ARF excitation was 1 kHz with a 50% duty cycle. At the two lower excitation intensities (one and two times the FDA limit for the mechanical index), we applied a continuous excitation for 10 seconds (10,000 pushes). At the two higher intensities, we applied 20 successive sequences of 125 pushes at 1 kHz with a duty cycle of 50% (2500 pushes). With these excitation parameters, the spatial peak temporal average intensity at the lowest ARF intensity was I_SPTA_ = 2.75 W·cm^−2^, 55 times above the FDA guidelines for the derated spatial peak temporal average intensity (I_SPTA.3_ ≤ 50 mW·cm^−2^). We recorded the displacement induced by the transducer with the SD-OCT system.

Immediately following the procedure, while the animals were still under anesthesia, both eyes were subject to a comprehensive ophthalmic evaluation in the animal suite using a surgical microscope equipped with slit-lamp illumination and the OCT system to assess the integrity of the cornea, anterior chamber, and lens. This evaluation utilized the Semi-quantitative Preclinical Ocular Toxicology Scoring (SPOTS) system for standardized assessment of ocular findings.^22^ The animals were then returned to their cages and observed until the effect of the anesthesia wore off. The animals were then returned to their cages in the animal facility under the care of the DVR.

On postoperative day 3 and on postoperative weeks (POWs) 1, 2, and 3, we performed a comprehensive ophthalmic evaluation using a slit-lamp and anterior OCT system on the awake rabbit to assess the integrity of the cornea, anterior chamber, and lens and measured the IOP under topical anesthesia with the same rebound tonometer. Upon completion of the final evaluation at POW 3, the animals were humanely euthanized. Euthanasia was performed via intravenous administration of a solution containing pentobarbital sodium and phenytoin sodium (390 mg/10 lb) into the marginal ear vein. Absence of vital signs was closely monitored to confirm death.

## Results

During our experiments, we observed no apparent damage to the rabbits’ eyes. Two of the rabbits exposed to ARF levels eight times the reference voltage developed mild localized conjunctival hyperemia immediately after exposure, corresponding to a score 1 on the SPOTS system, which resolved completely within 24 hours. Aside from this transient reaction, the ocular health of all rabbits, evaluated using slit-lamp examination and OCT, was found to be normal.

We observed lens displacements using the SD-OCT system at four and eight times the reference voltage, measuring 40 nm and 83 nm, respectively. Since the acoustic force is proportional to the intensity, we expect that the displacement amplitude at the intensity corresponding to the FDA limit will be at least several nanometers. However, displacements in the lens at one and two times the reference limit could not be discerned, possibly because the OCT system was not coaligned with the ARF probe in these experiments. We used a different positioning system for these experiments, which made alignment more difficult and prevented us from recording the deformations.

**Figure 3.**
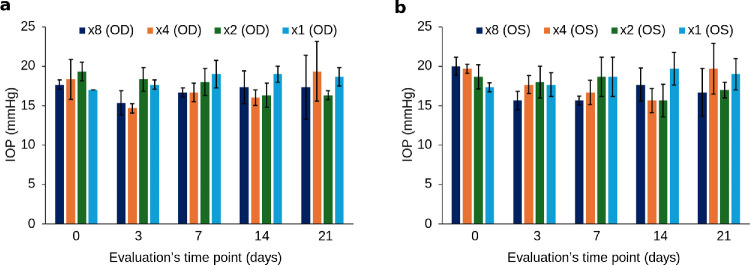
Intraocular pressure measured at each stage of the experiment. (**a**) Right eyes of rabbits exposed to ultrasound at eight, four, two, and one times the reference voltage. (**b**) Left eyes of the same rabbits, serving as controls.

**Figure 4.**
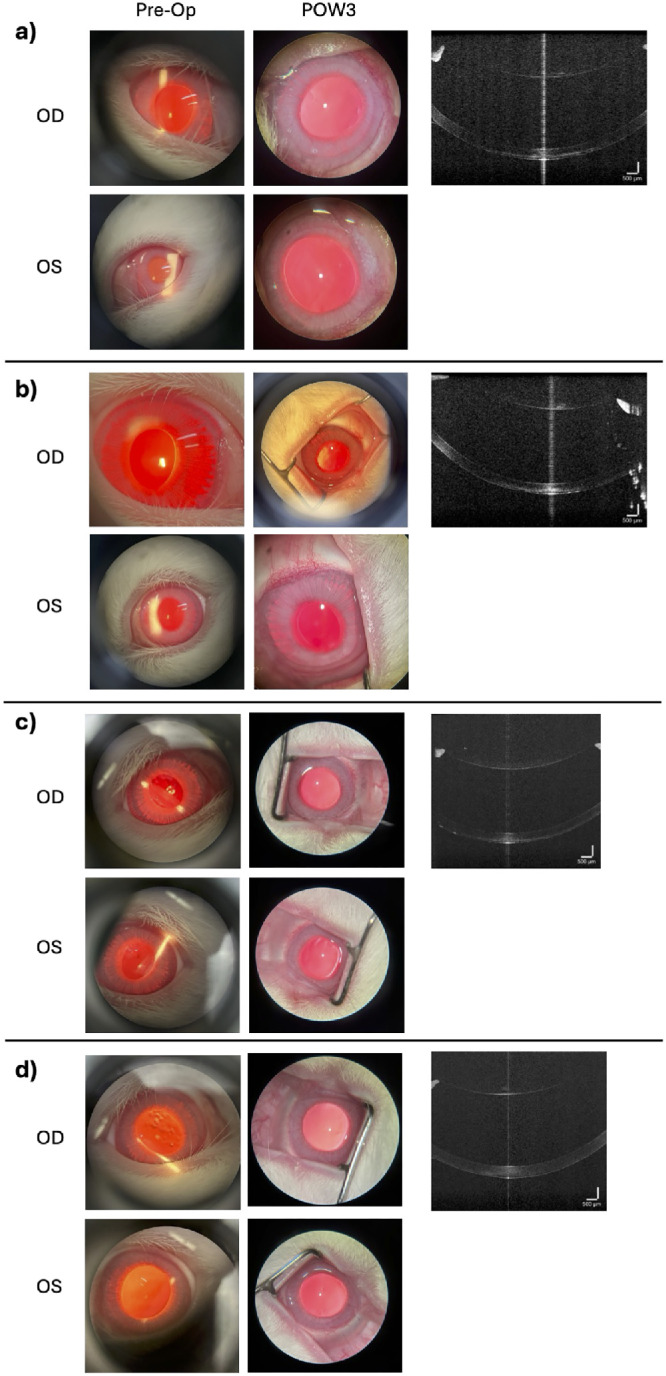
Slit-lamp and ophthalmic microscope pictures and anterior segment OCT images of rabbit eyes following ARF exposure at varying intensities (OD = exposed eye; OS = control eye). (**a**) One times the reference voltage. (**b**) Two times the reference voltage. (**c**) Four times the reference voltage. (**d**) Eight times the reference voltage. OCT images (*right column*) correspond to OD acquired at POW 3.

There was no significant difference in the IOP of the exposed eyes and the control eyes (Fig. 3). Throughout the follow-up period, no clinical or OCT-imaged abnormalities were observed in any group. Figure 4 displays images of both the right (treated) and left (control) eyes of a rabbit subjected to eight times the FDA reference voltage, as well as an OCT image of the right eye taken just before euthanasia.

## Discussion

We applied ARF to rabbit eyes, focusing particularly on the lens, and did not observe any sign of damage in the cornea, anterior chamber, and lens with acoustic intensities several times higher than the FDA-recommended limit.

The primary study endpoints were to demonstrate that ARF exposure of the lens does not induce cataract, anterior segment injury, or inflammation on clinical examination during a 3-week period following ARF application. These endpoints were selected because the ARF damage mechanism at high intensities involves structural damage due to the mechanical effects at high peak intensities and thermal damage due to excessive average intensity. These effects would lead to visible clinical effects, such as cataract or inflammation on clinical examination. We, therefore, used only clinical examination endpoints to determine safety and did not include histopathologic examination as an endpoint.

A transient mild bulbar conjunctival hyperemia was observed in two eyes exposed to the high level of intensity. Given that this occurred only in 2 of 12 eyes, we believe that it was caused not by the treatment itself but by the manipulation of the probe and potentially also an effect of the acoustic matching gel. In these two animals, it took longer to align and position the probe, which means that the probe was in contact with the eye for a longer period and may have caused transient mild irritation.

The displacement amplitude of the lens deformation at the two highest intensities (four and eight times the reference voltage) was on the order of 40 and 83 nm. Since the displacement is expected to be proportional to the intensity, we anticipate a displacement on the order of approximately 10 nm at the FDA limit for the MI, which is well above our detection threshold (∼1 nm). Together, the findings that ARF exposure at 8 times for MI and 14 times for I_SPPA_ the FDA limit induced no clinically observable tissue damage and that the displacement amplitude at the FDA limit is above our detection threshold support the feasibility of using ARF excitation for in vivo measurements of lens mechanical properties. On the other hand, our prior in vitro studies on intact porcine globes showed that the elastic wave velocity could be measured reliably using ARF exposures that satisfied the FDA limits for the peak intensity (I_SPPA_ = 24 W·cm^−2^), but not at the lower intensities corresponding to the FDA limit for the MI (I_SPPA_ = 6 W·cm^−2^). The difference in outcome with our present study could be due to differences between in vivo and in vitro conditions or to differences in species (rabbit versus pig). Further studies are needed to determine if measurable acoustic waves can be produced with ARF exposures that meet the FDA guidelines for the MI.

We could not measure the displacements at the two lowest intensities (one and two times the reference voltage) in the present study. A possible cause is that the OCT system was not coaligned with the ARF probe in these experiments, preventing us from recording the deformations. For these experiments, we used a different version of the positioning system, which was more difficult to coalign.

Note here that no derating of acoustic pressure was performed. The acoustic energy propagates through the acoustic gel, cornea, and aqueous humor to reach the lens. However, only the cornea has a high ultrasound attenuation and a thickness of approximately 500 µm, such that with a standard attenuation coefficient of 0.3 dB/MHz/cm, the pressure attenuation is insignificant.

With our ARF excitation parameters (1-kHz repetition rate with 50% duty cycle), the temporal average intensity exceeded the FDA guidelines (50 mW·cm^−2^) by 55 times at the lowest intensity and by 3872 times at the highest intensity. The temporal average intensity relates to the risk of thermal damage. In our application, the transducer is separated from the tissue by the large volume of gel contained in the cone. There is, therefore, a low risk of direct heating from the transducer surface itself.

It has been previously described that cataract formation after trauma can occur acutely within days or chronically after months or years.^23^ A study evaluating 33 cases of cataract formation after ocular trauma found that most of the cataracts reported were caused by blunt trauma (*n* = 18, 54.5% of the cases), followed by penetrating trauma (*n* = 14, 42.4% of the cases) and electric shock (*n* = 1). The average time lag between trauma occurrence and hospital presentation time due to cataract formation was 14.5 ± 35 days.^24^ In a separate experimental study on traumatic cataracts, Fagerholm and Philipson^25^ examined the quantitative changes in dry mass content following lens injury in 10 rabbits and 18 rats. Notably, they observed a thin posterior subcapsular opacity in 10 of the 18 rats within the first hour, while posterior subcapsular opacification was observed in rabbits 1 week after trauma. These findings underscore the variability in cataract formation onset following ocular trauma. In our study, none of the 12 rabbits developed any form of lens opacity during the 21-day follow-up period, suggesting that ARF may not cause acute damage to lens structures. However, it is important to acknowledge a potential limitation: our study's follow-up period was restricted to 21 days, and delayed cataract formation, akin to human traumatic cataracts induced by blunt trauma, may still occur over much longer periods. We also only conducted a single imaging session, and future work will focus on evaluating the eyes following serial imaging sessions.

Our study demonstrates that using ARF at levels 8 and 14 times the FDA-recommended limit for the mechanical index and the spatial peak pulse average intensity, respectively, does not lead to any noticeable damage to the lens. This is noteworthy, considering that previous studies have frequently used ARF at levels exceeding FDA guidelines to investigate the mechanical properties of ocular tissues ex vivo and in vivo.^12^^,^^13^^,^^26^^–^^28^

In conclusion, our study demonstrates that ARF exposure in vivo in a rabbit model with our delivery approach does not induce any clinical adverse effects even at intensities that are 8 to 14 times higher than the FDA-recommended limit. Based on this finding, we conclude that ARF can be a suitable approach for lens elastography in the human eye if the system has sufficient sensitivity to detect displacement induced at intensities below the FDA guidelines for diagnostic ultrasound.
